# An informatics research platform to make public gene expression time-course datasets reusable for more scientific discoveries

**DOI:** 10.1093/database/baaa074

**Published:** 2020-11-28

**Authors:** Braja Gopal Patra, Babak Soltanalizadeh, Nan Deng, Leqing Wu, Vahed Maroufy, Canglin Wu, W Jim Zheng, Kirk Roberts, Hulin Wu, Ashraf Yaseen

**Affiliations:** Department of Biostatistics and Data Science, School of Public Health,The University of Texas Health Science Center at Houston, 1200 Pressler Street, Houston, TX 77030, USA; Department of Biostatistics and Data Science, School of Public Health, The University of Texas Health Science Center at Houston, 1200 Pressler Street, Houston, TX 77030, USA; Department of Biostatistics and Data Science, School of Public Health, The University of Texas Health Science Center at Houston, 1200 Pressler Street, Houston, TX 77030, USA; Department of Biostatistics and Data Science, School of Public Health, The University of Texas Health Science Center at Houston, 1200 Pressler Street, Houston, TX 77030, USA; Department of Biostatistics and Data Science, School of Public Health, The University of Texas Health Science Center at Houston, 1200 Pressler Street, Houston, TX 77030, USA; TechWave International. Inc., Houston, TX, USA and; School of Biomedical Informatics, The University of Texas Health Science Center at Houston, 7000 Fannin St. Suite 600, Houston, TX 77030, USA; School of Biomedical Informatics, The University of Texas Health Science Center at Houston, 7000 Fannin St. Suite 600, Houston, TX 77030, USA; Department of Biostatistics and Data Science, School of Public Health, The University of Texas Health Science Center at Houston, 1200 Pressler Street, Houston, TX 77030, USA; School of Biomedical Informatics, The University of Texas Health Science Center at Houston, 7000 Fannin St. Suite 600, Houston, TX 77030, USA; Department of Biostatistics and Data Science, School of Public Health, The University of Texas Health Science Center at Houston, 1200 Pressler Street, Houston, TX 77030, USA

## Abstract

The exponential growth of genomic/genetic data in the era of Big Data demands new solutions for making these data findable, accessible, interoperable and reusable. In this article, we present a web-based platform named Gene Expression Time-Course Research (GETc) Platform that enables the discovery and visualization of time-course gene expression data and analytical results from the NIH/NCBI-sponsored Gene Expression Omnibus (GEO). The analytical results are produced from an analytic pipeline based on the ordinary differential equation model. Furthermore, in order to extract scientific insights from these results and disseminate the scientific findings, close and efficient collaborations between domain-specific experts from biomedical and scientific fields and data scientists is required. Therefore, GETc provides several recommendation functions and tools to facilitate effective collaborations. GETc platform is a very useful tool for researchers from the biomedical genomics community to present and communicate large numbers of analysis results from GEO. It is generalizable and broadly applicable across different biomedical research areas. GETc is a user-friendly and efficient web-based platform freely accessible at http://genestudy.org/

## Introduction

Over the past few decades, substantial funding and resources have been invested to generate biomedical datasets at many levels, ranging from nucleic acid and gene level to population level, in order to understand, treat and prevent various diseases, and protect public health. Based on data sharing policies of National Institute of Health (NIH) and other government agencies, many of aforementioned datasets are required to be shared with the general research communities. Consequently, vast amounts of biomedical data are being accumulated in databases and data repositories. However, use or reuse of these existing datasets for research by third parties is still not common as expected.

Gene expression data from various diseases under different experimental conditions are mostly deposited in the NIH/NCBI-sponsored Gene Expression Omnibus (GEO) data repository (1). Like many of the biomedical databases, GEO was originally created as a data repository to comply with the data sharing policies. Often, these data sharing platforms are designed and organized for easy and convenient data submission by experimentalists, but not friendly for data retrieval and analysis. Further, it is not easy to identify the particular datasets to address a particular biological question for a specific disease from GEO, since the experimental design and study description are documented in an unstructured free text. Hence, it is necessary to use text mining and natural language processing (NLP) technologies to restructure the existing repository so that datasets can be findable, accessible and reusable.

This article describes a web-based platform that addresses the difficulties in finding, accessing, reusing biomedical datasets, specifically from GEO, as well as the difficulties in finding and forming collaborations. The novel platform, named as Gene Expression Time-Course Research (GETc) platform (http://genestudy.org/), is built on top of an analytical method based on the ordinary differential equation (ODE) model for analyzing time-course gene expression data. GETc offers the following services and functions:

- Hosts time-course gene expression datasets from GEO annotated with disease and cell types.
- User-friendly navigation and searching functions.
- Hosts analysis results of the time-course gene expression datasets produced by the ODE analytic pipeline.
- Recommends relevant datasets for users based on their research interests.
- Recommends relevant papers and collaborators for each dataset hosted in the platform.

The rest of the article is organized as follows: Section 2 discusses the background of the analytic pipeline and recommendation systems. Section 3.1 presents datasets used for developing the GETc platform. Section 3.2 describes the methodology used for analytic pipeline, recommendation systems and platform implementation. Section 4 describes and discusses the results. Finally, conclusions are presented in Section 5.

## Background

In this section, we present the three main parts of our work, (i) repositories developed for archiving datasets in the biomedical domains and their metadata, (ii) an analytic pipeline developed for analyzing gene data and (iii) dataset, literature and collaborator recommendation systems.

### Dataset repositories

It is a growing trend among the researchers to make their data publicly available for reproducibility and data reusability. Many repositories and knowledge bases have been established for different types of data in many doma-ins. GEO(www.ncbi.nlm.nih.gov/geo/), UKBioBank(www.ukbiobank.ac.uk/), ImmPort(www.immport.org/home) and TCGA(portal.gdc.cancer.gov) are a few examples of repositories in the biomedical domain. DATA.GOV archives the U.S. Government’s open data from agriculture, climate, education, etc. for research use. However, users from the biomedical community have to visit and search each repository separately to find data for their research, which can be time-consuming and hectic.

DataMed(datamed.org) started an initiative to solve the above issue for the biomedical community by combining biomedical repositories and enhancing the query searching using advanced NLP techniques (2, 3). DataMed indexes and searches diverse categories of biomedical datasets (3). DataCite is another data discovery index, which includes 16 187 835 works from many different domains (4). However, these repositories do not provide either insight of data or help to find collaborators, which are still challenging tasks to accomplish.

### Analytic pipelines for gene expression data

The study of gene regulation related to different biological functions is critical to understand the underlying mechanism of each function, such as cell growth, division, development and response to environmental stimulus. In addition, gene regulatory networks (GRN) have been shown useful for investigating the interaction among genes involved in a biological process, or genes responsive to an external stimulus. There are many computational approaches in the literature for inferring GRNs from gene expression data; for example, information theory models (5–7), Boolean networks (8–11) and Bayesian networks (12–15). However, these approaches are either not efficient in describing dynamic regulations between genes or restricted to small-scale networks. Meanwhile, responses to environmental stimulus, such as immune response to viral infection or response to aberrant activation of a particular pathway, are dynamic processes and require deliberate analysis of time-course gene expression data, which in turn is an ultra-high dimensional problem and needs the use of advanced statistical and computational approaches developed. Therefore, we implement an alternative comprehensive approach that exploits ODE models and gene regulatory network analysis developed in (16–18). This model takes into account the dynamic and temporal behavior of genes, and learns the dynamic relation between genes, in the form of stimulator or inhibitor of each other. Genes (or probes) with significant expression level changes over time are identified as dynamic response genes. Then the top 3000 dynamic response genes are clustered into groups according to their expression pattern over time. Finally, a regulatory network is established by the ODE model (19).

### Recommendation systems

A recommendation system is an enabling mechanism to overcome information overload. Literature in this area can be broadly grouped as content-based or collaborative filtering based recommendation systems. Next, we discuss literature related to developed recommendation systems.

#### Dataset recommendation

There are many dataset repositories in the biomedical domain and many datasets are added to each repository on a daily basis. For example, 34 datasets were added to GEO repository daily in 2019. Hence researchers are likely to be overwhelmed with the data available and they have to visit each repository for searching a dataset. The platforms like DataMed solved this problem and researchers only had to visit DataMed for searching the datasets. However, DataMed has not been updated recently. Again, the intent of search is always difficult to identify (20). A dataset recommendation system based on researcher’s profile may be helpful for information filtering. There were a few experiments performed on data linking (21–23) where similar datasets were clustered together using different semantic features. Most of these works were on linking the datasets with similar datasets rather than a dataset recommendation.

#### Literature recommendation

The usefulness of the literature recommendation can be stated by the acceptance of Google Scholar, Semantic Scholar, PubMed, etc. The CiteSeer project (24, 25) was the first of its kind to start research paper recommendation. Later, many scientific article recommendation systems were developed. Science Concierge is a content-based article recommendation system using distributional semantics (LSA) and the relevance feedback (Rocchio algorithm). It recommends articles for any number of input articles based on the 2015 Society of Neuroscience Conference articles (26). (27) proposed a citation-based collaborative filtering recommendation system for research articles using Jaccard similarity. Similar article recommendation systems have been developed using TF-IDF (28), topic modeling (29) and citation or author network analysis (30). TF-IDF was the most frequently applied weighting scheme for recommendation tasks (25).

SciMiner is a web-based platform for identifying gene names in text based on user input and provides literature from MEDLINE for the corresponding gene (31). A content-based PubMed article recommendation system, PURE, was developed using Expectation Minimization (32) and it recommends articles to users based on their preferred articles. (33) developed a probabilistic topic-based model for content similarity called ‘*pmra*’ on the publications from MEDLINE and this has been used as a related article search function in PubMed. Most of the proposed literature recommendation systems use embedding methods to convert text into vectors and calculate the similarity between articles.

Once a researcher finds a dataset suitable for his/her study, he/she may need literature available related to the dataset. A literature recommendation system for datasets may be a helpful tool for this scenario where researchers can get literature from PubMed for each dataset.

#### Collaborator recommendation

Academic collaborator recommendation has long been regarded as a useful application in the academic environment, which aims to find potential collaborators for a given researcher by exploiting big academic data. In the past few years, several works on collaborator recommendation have been proposed (34–37).

Mainly, co-author network information has been incorporated to enhance the collaboration recommendation (35, 37, 38). (38) proposed a random walk restart model on co-author order, latest collaboration time point and collaboration times. (37) developed a collaborator recommendation system using collaborative entity embedding developed using the topic words collected from the publications of researchers. The cross-domain collaborator recommender is another important aspect of this recommendation and (36) proposed a cross-domain collaborator recommendation using the co-author matching, topic matching and cross-domain topic learning.

(35) proposed CollabSeer based on the co-author network and lexical similarity. However, it is difficult for new researchers or students to get recommendation using the co-author network or lexical similarity as they do not have papers. (39) proposed a collaborator recommender for new researchers or students using input keywords, organizational relationship, ratings and activity level of the collaborators.

When a researcher finds suitable data for his/her study, the researcher may look for collaborators to work with on that dataset. In this scenario, a collaborator recommendation system for each dataset may be helpful.

## Materials and methods

### Data

#### GEO Metadata collection

GEO is one of the most popular public repositories for functional genomics data. As of 18 December 2019, there were 122 222 series of datasets available in GEO. Metadata of GEO datasets such as title, summary, date of publication and name of authors was collected from the GEO using a web crawler. The PMIDs of the articles associated with each dataset were also collected. Many datasets did not have associated articles.

**Time-course dataset**: This study was conducted for the time-course datasets from GEO, however, the time-course datasets were not identified explicitly in the GEO websites. The time-course datasets can be identified manually by reading the dataset descriptions or scanning the associated data with it which is a time-consuming and tedious task. A keyword-based NLP method was applied for identifying time-course datasets. We implemented a regular expression-based approach to extract the time point information from the GEO metadata. For example, some phrases like ‘12 time points’, ‘7 developmental stages; harvest at 10 hrs, 12 hrs’, etc. were used to extract the time point information. The regular expression-based system was able to identify 167 datasets out of 200 random datasets with an accuracy of 83.5%. Further, a total of 555 datasets were filtered manually from 862 datasets identified by the above system for processing. More details on identifying time-course datasets can be found in (40). Once the datasets are identified, the GSE number were fed to the pipeline (Section 3.2.1) and it automatically retrieved the data and metadata information corresponding to GSE numbers. In addition to the time points, diseases, organisms or/and cell types were identified from the title and summary of the datasets. MetaMap (41) applied to the metadata, and the Human Disease Ontology (DOID) terms were detected from the annotated text for each dataset (42). Further, datasets can be filtered using both the cell type and diseases.

#### MEDLINE Articles

For developing dataset recommender, we collected the researcher’s publications from PubMed. MEDLINE articles were collected for developing literature and collaborator recommenders. MEDLINE articles were collected from PubMed which comprises more than 29 million biomedical and life science research articles. These articles consist of information such as title, abstract, authors, affiliations, Medical Subject Headings (MeSH) terms and publisher name.

However, the articles collected from PubMed contain a variety of topics related to biomedicine and life sciences which may not be suitable for building a recommendation system for datasets in GEO. Further, the articles before 1998 were removed as the research on micro-array data started during that year (43). The datasets that are related to gene expressions and articles collected from PubMed contain a variety of topics. Thus, a MeSH term-based filtering method was implemented to remove unrelated articles from the whole MEDLINE articles. The details of the filtering method can be found in (43). A total of 770 537 articles were utilized for developing literature and collaborator recommendations.

### Methods

#### Analytic pipeline for time-course gene expression data

We integrated the series of statistical and modeling methods for the time-course gene expression data into an analytic pipeline (19) which includes eight steps as mentioned in Figure 1.

**Figure:**
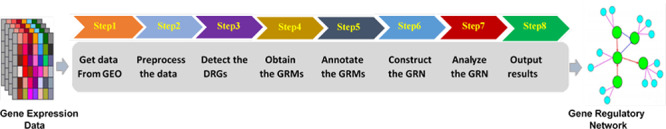
**Figure 1.** Time-course gene expression analytic pipeline.

The final analysis results of the pipeline can be reported as the initial bioinformatics findings for narrowing down the analysis and framing scientific questions, toward new collaborative publications. We could apply the pipeline to each of the time-course gene expression datasets under one experimental or biological condition. Furthermore, simple comparison functions between two or more datasets across experimental conditions and/or from different studies are currently under development for the pipeline. We published the source code of the analytic pipeline, so others can use the pipeline and expand its functionalities.(github.com/j142857z/Pipeline (Original code)),(github.com/AutumnTail/Pipeline (Updated code)).

#### Recommendation systems

**Data Recommendation:** Data recommendation is an essential part of the GETc platform. The dataset recommendation function recommends datasets to researchers based on their publications. The datasets used for this recommendation system contain data not only from GEO but also from other sources such as TCGA, ArrayExpress, SRA and Clinical Trails. We used only textual information of datasets (title and summary) and publications (title and abstract).

A researcher may have multiple research interests. To identify the research interests, we implemented a non-parametric clustering algorithm named Dirichlet process mixture model (DPMM). More details on DPMM and its parameter tuning for obtaining better number of clusters can be found in (44). Each researcher had to provide name and curriculum vitae (CV)/list of publications to get dataset recommendation. Researcher’s names were searched in PubMed to get publications (title, abstract, year of publication). This search may result publications from other researchers with the same name which was solved by searching the title of the publication from PubMed in the CV/list of publications provided by the researcher. Finally, publications of the authors were clustered using DPMM to obtain the research topics. For each topic, datasets can be recommended by calculating cosine similarity of research field/cluster vector and dataset vectors. The detailed methodology and evaluation can be found in our previous publication on dataset recommendation (44).

**Literature Recommendation:** The literature recommendation system recommends literature for datasets. The most similar literature for a dataset can be determined simply by comparing the cosine similarity of the dataset vector and paper vectors. For developing the literature recommendation system in GETc, we used BM25 as it resulted in better precision at 10 compared to other embedding methods such as TF-IDF, word2vec and doc2vec (43). Finally, we used the title based weighted re-ranking and text normalization methods to improve the retrieved results. The detailed methods, experiments and results can be found in our previous publication (43).

**Collaborator Recommendation:** For each dataset, the recommendation system suggests some collaborators based on the recommended literature. We can say that the authors of the top similar literature for a dataset can be suitable collaborators to work with on that dataset. The authors of the similar articles may have experience working on the dataset and already published articles using it. Further, the collaborators may be recommended for each dataset by ranking the unique authors of the retrieved similar articles. For a dataset (*d*), the score for each unique author of similar articles can be calculated using Equation (1).

(1) \begin{equation*} \textrm{AuthorScore}_{i} = \sum_{j=0}^n \textrm{SimScore}_{j} * \textrm{weight} \end{equation*}

$$\begin{equation*} \textrm{weight} = \begin{cases} 0\ \textrm{if } A_i \notin P_j\\ 1\ \textrm{if } A_i\,\,\,\textrm{is the first or last author in } P_j \\ 0.1\ \textrm{if } A_i\,\,\,\textrm{is not first or last author in } P_j \end{cases} \end{equation*}$$

where AuthorScore_*i*_ is the score for *i*th author calculated over all the retrieved similar articles ($P = {P_0, P_1, ...P_n}$) for *d*. *n* is the number of total retrieved article for *d*. SimScore_*j*_ is the similarity score of *d* and *j*th article (*P*_*j*_).

Higher weights were provided to the first and last authors of each similar article whereas less weights were provided to all other authors. Finally, the authors with the highest scores were recommended as the collaborators for *d*.

The top 1000 recommended publications from the above literature recommender for a single dataset were used for identifying collaborators for that dataset. Furthermore, authors’ affiliations provided in papers were parsed using the *affiliation_parser*(github.com/titipata/affiliation_parser) package and the distance between the recommended collaborators’ and the user’s current location was calculated using *geopy*(geopy.readthedocs.io) package to show a distance-based relevance of user and collaborators.

### GETc Platform

In this work, we developed an interactive web-based platform, called GETc, to facilitate collaboration and sharing of the analytic results of our pipeline on time-course gene expression data from GEO to the general research community. We have identified 555 time-course gene expression datasets with more than 7 time points from GEO. We applied our analytic pipeline on 37 of those datasets (results in Section 4). The output of the analytic pipeline for each dataset is folder of files containing intermediate and final analytic results, tables, graphics/plots and documents. The output also includes an automatically generated analysis report for each dataset.

Platform users could interactively search, browse and identify particular datasets and corresponding results of interest. They can visualize and review the analysis results including figures and tables, which can be easily downloaded via the platform web-based user-interface. For the unprocessed time-course gene expression datasets included in the platform, users can request to execute the pipeline. The platform also provides its users with recommendations by employing the recommendation systems described in Section 3.2.2. It recommends literature for time-course gene expression datasets, potential collaborators for extracting scientific insights from the analytic results. It also recommends datasets to researchers. Figure 2 shows the overview of GETc platform. GETc platform executes the tasks mentioned inside the green box.

**Figure:**
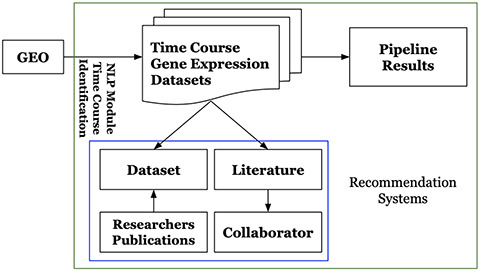
**Figure 2.** High-level architecture of the GETc platform.

**Figure 3.**
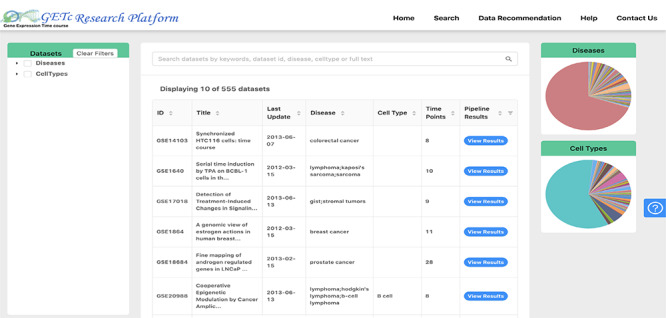
Search and view datasets in GETc research platform.

Users of the platform can search for a time-course dataset using keywords and phrases and see the literature available, significant gene lists, gene clusters and prospective collaborators for that dataset. A screenshot of search and view dataset functionalities is shown in Figure 3. The dataset can be searched if any of the searched keywords matched with the dataset id, title, abstract or platform organism. The datasets retrieved can be filtered using disease or cell type provided on the left side tree view or right side pie charts. The disease types are extracted from human disease ontology (40).

## Results and discussion

The results of the analytic pipeline which we applied on 37 time-course gene expression cancer datasets from GEO are presented in Table A1. For each dataset with different conditions, the table shows the number of DRGs, number of GRMs, number of time points, cancer type, cell line, the organism, vitro or *ex vitro* or *in vitro* or *in vivo* and species (human or mouse/rats species). MCF10A, MCF7, HeLa and other widely used cell lines are tested in these datasets. These cells lines are originated from various types of cancers such as breast cancer, cervical cancer and leukemia. Also, treatments in these datasets target several essential cancer pathways, such as NFkB, EGFR and hedgehog. These classifications will help researchers perform meta-analyses to identify common/key genes and GRN in a certain type of cancer.

Evaluating recommendation systems are challenging because no benchmark nor prior true annotation exists for either dataset recommendation or dataset-driven literature recommendation. For that reason, we performed a manual evaluation by asking expert human judges to rate the recommendation of systems using one to three ‘stars’ scale based on the relevance (1: not relevant, 2: partially relevant, 3: most relevant).

We evaluated the recommendation systems using strict and partial precision at 10 (P@10). Strict considers only 3-star, while partial considers both 2- and 3-star results. The developed dataset recommendation system was evaluated with five judges who have worked on the datasets before. The system obtained P@10 (strict) and P@10 (partial) of 0.61 and 0.78, respectively. For the literature recommendation, we considered 36 datasets for evaluation and the human judges have already worked on these datasets earlier. The proposed system obtained 0.80 and 0.87 of P@10 (strict) and P@10 (partial), respectively.

No gold standard dataset for evaluating collaborator recommendation is available to date. Similar to literature recommendation, evaluating our collaborator recommendation system was a challenging task, as it requires time to work with collaborators and only then they can provide feedback for system’s output. We are currently working with additional multiple collaborators to evaluate the output of the system and generate feedback that we can use to assess the system’s quality in the future.

A screenshot of literature (top right corner) and collaborator (bottom right corner) recommendations for dataset GSE14 103 is provided in Figure 4. For a selected dataset on the platform UI, the literature recommendation system will generate a list of related papers recommended for users. The recommended list of collaborators can be sorted by name or distance. We have a plan to implement a search function which will allow users to search for collaborators based on the preferred city.

**Figure:**
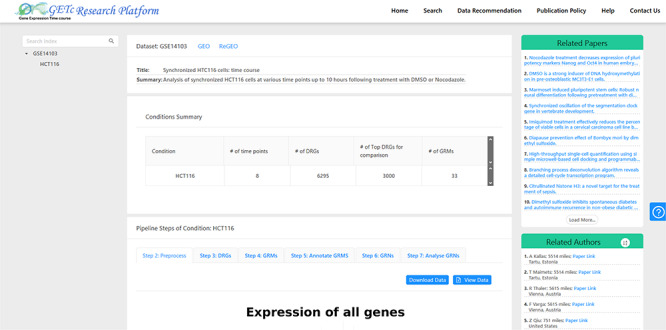
**Figure 4.** A screenshot of recommended literature and collaborators for GSE14103.

We believe the functions of GETc are very useful for researchers from the biomedical genomics community to present and communicate large numbers of analysis results. In addition to datasets from GEO, we are currently expanding the platform with new time-course datasets from other repositories such as TCGA, SRA and ImmPort. We applied the ODEs in the process of constructing the high-dimensional gene regularity network where having at least 8-time points was essential for the identifiability of the corresponding model. Thus, only datasets with more than or equal to 8-time points can be processed with our pipeline.

## Conclusion

In this work, we developed a novel research platform called GETc for sharing data and analytic results of time-course gene expression datasets from GEO to improve the dataset reusability. It is built on top of an analytical method based on the ODE model for analyzing time-course gene expression data. GETc platform provides means to efficiently search and retrieve data, results, and facilitate collaboration through recommendation of related literature and potential collaborators corresponding to datasets. This platform also hosts a dataset recommendation system which will help researchers in biomedical domain to search datasets based on their publications. This will hopefully lead to better data reuse experience. We believe that the proposed novel idea and computational platform could also be applied to other types of data from different databases or data repositories.

## Appendix

**Table A1.** Results statistics from the cancer datasets (ORG: Organism, *in vitro*: ivr, *ex vitro*: evv, *in vivo*: ivv, Species: SP, Homo sapiens: HS, Rattus norvegicus: RN, Mus Musculus: MM)

SL	GEO accession	Time point	Cancer type	Cell line	ORG	SP	Condition	# of DRG	# of GRM
1	GSE1864	11	Breast cancer	ZR-75.1	ivr	HS	17*β*-estradiol	1272	85
							17*β*-estradiol (dye swap)	1145	34
2	GSE3113	8	Colorectal carcinoma	EcR-RKO/KLF4	ivr	HS	Ponesterone	3349	44
							Control	1589	50
3	GSE770	10	Prostate adenocarcinoma	LNCaP C4-2	ivr	HS	Irradiation	4227	72
4	GSE1640	10	Kaposi’s sarcoma	BCBL-1	ivr	HS	Cidofovir rep1	473	146
							Cidofovir rep2	453	137
							Cidofovir rep3	301	164
							Control rep1	643	125
							Control rep2	504	121
							Control rep3	568	142
5	GSE9048	14	N/A	Embryonic stem	ivr	MM	HDRep1	21 349	30
							HDRep2	21 349	37
							HD_LIF	20 209	36
6	GSE9854	10	Osterosarcoma	U2OS	ivr	HS	GFP	6400	84
							HIC1	7062	61
7	GSE14103	8	Colorectal carcinoma	HCT116	ivr	HS	Nocodazole	6295	33
8	GSE17018	9	stomach	GIST-T1	ivr	HS	Imatinib mesylat Rep1	13 121	42
		9					Imatinib mesylat Rep2	13 121	42
		8					Imatinib mesylat Rep3	23 002	34
9	GSE20361	8	Breast cancer	MCF-7	ivr	HS	17*β*-estradiol		20
10	GSE20988	8	Mediastinal (thymic) large B-cell lymphoma	K1106	ivr	HS	JAK2 inhibitor	4766	46
11	GSE22955	16	Breast cancer	SUM-225	ivr	HS	HER-2 inhibitor CP724,714	11 725	84
12	GSE23135	16	Breast cancer	MCF-10A	ivr	HS	Gfitinib	10 046	50
13	GSE23136	16	Breast cancer	MCF-10HER-2	ivr	HS	Gfitinib	12 184	49

**Table A1.** (Continued)

SL	GEO accession	Time point	Cancer type	Cell line	ORG	SP	Condition	# of DRG	# of GRM
14	GSE18684	28	Prostate adenocarcinoma	LNCaP	ivr	HS	R1881_Rep1-1	8666	41
		28					R1881_Rep1-2	9293	121
		9					R1881_Rep2-1	4272	49
		9					R1881_Rep2-2	4052	51
15	GSE21618	8	Breast cancer	MCF-7	ivr	HS	TamR_Control	6676	47
							TamR_E2	4859	38
							TamR_E2_Tamoxifen	11 314	36
							TamR_HRG	14 764	38
							TamR_HRG_Tamoxifen	10 243	31
							TamR_Tamoxifen	10 345	35
							WT_E2	7606	35
							WT_E2_Rep1	8619	39
							WT_E2_Rep2	3267	41
							WT_E2_Tamoxifen	6059	37
							WT_HRG	8370	34
							WT_HRG_Rep1	11 724	32
							WT_HRG_Rep2	9274	42
							WT_HRG_Tamoxifen	6093	35
							WT_Tamoxifen	3530	37
16	GSE41072	19	Acute T cell leukemia	Jurkar or Primary T cells	ivr	HS	Jurkat Roc	14 382	44
		12					Tcell Roc	8520	59
17	GSE26002	8	Glioblastoma	TRP mouse model	ivv	MM	TRPhet	1328	45
18	GSE38623	13	Skin cancer	Mouse whole back skin	evv	MM	UVB	11 225	104
19	GSE29641	8	Breast cancer	DU145; HT29; MCF7	ivr	HS	Hypoxia	6325	26
							Hypoxia	7651	29
							Hypoxia	8169	29
20	GSE41034	8	Diffuse large B-cell lymphoma	HBL-1	ivr	HS	IkB kinase beta inhibitor MLN120B	15 278	43

**Table A1.** (Continued)

SL	GEO accession	Time point	Cancer type	Cell line	ORG	SP	Condition	# of DRG	# of GRM
21	GSE23137	16	Breast cancer	MCF-10HER-2	ivr	HS	HER-2 inhibitor CP724,714	11 469	140
22	GSE23138	16	Breast cancer	MCF-10A	ivr	HS	HER-2 inhibitor CP724,714	8811	120
23	GSE23139	16	Breast cancer	MCF-10HER-2/E7	ivr	HS	HER-2 inhibitor CP724,714	9221	96
24	GSE32869	11	Pancreas adenocarcinoma	AR42J	ivr	RN	Gastrin	7181	81
		12					Control	6594	92
		11					Gastrin	5515	105
		12					Control	6282	144
25	GSE41491	8	Breast cancer	DU145; HT29; MCF7	ivr	HS	Hypoxia	6127	27
							Hypoxia	7406	30
							Hypoxia	8011	24
26	GSE44700	12	B-cell Precursor leukemia cell line	BLaER1	ivr	HS	E2 treatment rep1	31 583	48
							E2 treatment rep2	23 767	68
27	GSE46045	14	Desmoplastic cerebellar medulloblastoma	Daoy	ivr	HS	Control_median	7176	216
							EGF_median	15 659	48
							EGF_SHH_median	17 972	51
							SHH_median	10 770	237
28	GSE49583	8	Pancreatic carcinoma	Primary pancreatic stellate cells	ivr	HS	Tumor-cell supernatant	4469	48
29	GSE49584	8	Pancreatic carcinoma	MiaPaca2	ivr	HS	Control	5441	44
30	GSE49586	9	Pancreatic carcinoma	MiaPaca2	ivr	HS	Stellate-cell supernatant	14 601	37
31	GSE50624	8	Acute T cell leukemia	Jurkat	ivr	HS	CDK7 inhibitor	30 013	9
							CDK7 inhibitor	29804	13

**Table A1.** (Continued)

SL	GEO accession	Time point	Cancer type	Cell line	ORG	SP	Condition	# of DRG	# of GRM
32	GSE52710	10	Hodgkin lymphoma	L428	ivr	HS	LNA-antimiR-9	3921	56
							LNA-Scrable	1732	66
33	GSE15327	9	Non-small cell lung cancer	NCI-H1975	ivr	HS	H2O2	2446	75
							Menadione	8912	24
34	GSE50988	23	Osteosarcoma	U2OS	ivr	HS	Thymidine-nocodazol	7763	792
		20					Thymidine rep1	18 894	166
		24					Thymidine rep2	9593	390
		24					Thymidine rep3	24 583	199
35	GSE64073	17	Breast cancer	MCF7	ivr	HS	DHMEQ	20	233
		16					HRG	15533	102
		16					HRG + DHMEQ	16 573	62
		16					HRG + LY294002	12 128	174
		17					LY294002	14 309	48
		17					Control	6427	193
36	GSE71721	11	Burkitt lymphoma	Primary lymphoma	evv	HS	anti human IgM F(ab)2 fragment rep1	6294	58
		10					anti human IgM F(ab)2 fragment rep2	4479	62
		10					anti human IgM F(ab)2 fragment rep3	4479	62

**Table A1.** (Continued)

SL	GEO accession	Time point	Cancer type	Cell line	ORG	SP	Condition	# of DRG	# of GRM
37	GSE15523	8	Skin cancer	BJ NMyc	ivr	HS	N-MycER(delta-MbII)	3875	44
							N-MycER	2741	54
38	GSE17708	9	Lung adenocarcinoma	A549	ivr	HS	TGFb1	20 296	57
39	GSE18817	8	Diffuse large B-cell lymphoma	HBL-1	ivr	HS	MLN120B	11 865	51
40	GSE34228	26	Lung adenocarcinoma	PC9	ivr	HS	Gefitinib	30 565	73
41	GSE21245	10	Pancreatic adenocarcinoma	LNCaP	ivr	HS	Dihydrotestosterone miRNA array	143	188
							Dihydrotestosterone miRNA array	13 636	93
42	GSE34243	17	N/A	Pgk12.1	ivr	MM	Differentiation induction	343 738	49
43	GSE45958	8	Breast cancer	Control	ivr	HS	2gy Radiation	56 560	44
							6gy Radiation	27191	46
							R6gy	43 650	46
44	GSE76368	8	Breast cancer	MCF-7	ivr	HS	Starvation	3229	51
45	GSE84096	11	Non-small cell lung cancer	NCI-H1975	ev	HS	EGF	9443	90
		8					Control	7059	64
